# Micro-Eukaryotic Diversity in Hypolithons from Miers Valley, Antarctica

**DOI:** 10.3390/biology2010331

**Published:** 2013-02-22

**Authors:** Jarishma K. Gokul, Angel Valverde, Marla Tuffin, Stephen Craig Cary, Don A. Cowan

**Affiliations:** 1 Institute for Microbial Biotechnology and Metagenomics, University of the Western Cape, Cape Town, Bellville 7535, South Africa; E-Mails: jk.gokul@gmail.com (J.K.G.); marlatuffin@gmail.com (M.T.); 2 Centre for Microbial Ecology and Genomics, Department of Genetics, University of Pretoria, Pretoria 0002, South Africa; E-Mail: angel.valverde@up.ac.za; 3 The International Centre for Terrestrial Antarctic Research, Department of Biological Sciences, University of Waikato, Private Bag 3105, Hamilton 3240, New Zealand; E-Mail: caryc@waikato.ac.nz

**Keywords:** Antarctica, micro-eukaryotes, hypoliths

## Abstract

The discovery of extensive and complex hypolithic communities in both cold and hot deserts has raised many questions regarding their ecology, biodiversity and relevance in terms of regional productivity. However, most hypolithic research has focused on the bacterial elements of the community. This study represents the first investigation of micro-eukaryotic communities in all three hypolith types. Here we show that Antarctic hypoliths support extensive populations of novel uncharacterized bryophyta, fungi and protists and suggest that well known producer-decomposer-predator interactions may create the necessary conditions for hypolithic productivity in Antarctic deserts.

## 1. Introduction

Microbial life in terrestrial Antarctica soils is subjected to extreme low temperatures, low water availability, high salinity, high UV radiation, and low nutrient availability [1]. However, despite the many adverse environmental constraints this extreme ecosystem has been shown to support extensive microbial biomass [2].

Much of the microbial research in Antarctic terrestrial and aquatic ecosystems has focused on the bacterial populations, and to a lesser extent on the archaea [3,4] and viruses [5,6]. In contrast, eukaryotic microorganisms have received much less attention [7].

Hypolithic communities in the Dry Valleys region of eastern Antarctica colonize the ventral surface of quartz rocks at the rock-soil interface [8,9,10,11]. Hypoliths can be envisioned as a stress-avoidance strategy, where the overlying rock creates a favorable sub-lithic microhabitat with greater physical stability, increased water availability, desiccation buffering, and UV protection [9,12]. As they are typically dominated by cyanobacteria [8,13] or bryophytes [9], hypolithons represent an important contribution to regional productivity [14,15]. Fungal dominated hypoliths have also been described in Antarctica [9].

In an earlier study [11] we characterized the bacterial and eukaryotic phylogenetic diversity of two hypolith types: Type I (cyanobacteria-dominated) and Type III (moss-dominated). Here, we extend this research to Type II hypolithons (fungal-dominated) with a focus on the micro-eukaryotic communities. We also compare all three types of eukaryal communities in terms of habitat preferences.

## 2. Results and Discussion

Environmental DNA was used as template for construction of three separate clone libraries using universal 18S rRNA, 18S-28S rRNA (ITS), and microalgal 18S rRNA-specific PCR primers (Table 1). A total of 31 unique phylotypes was found (Table 2). Most of the sequences showed low identity values, indicating that the majority of sequences might represent novel taxa. Rarefaction curves (not shown) showed that more extensive sequencing would be required to capture the complete diversity within micro-eukaryotic communities in hypoliths. Incomplete sampling might be aggravated by the inherent limitations of the PCR approach, since several groups of micro-eukaryotes (e.g., multinucleated fungi) have multiple rRNA gene copy numbers that would be preferentially amplified because of primer competition [16].

Phylotypic analyses demonstrate that diverse communities of micro-eukaryotes inhabit Antarctic hypoliths (Table 2), showing both a broad range of taxa and a large functional diversity, including phototrophs (bryophyta) and a variety of heterotrophic organisms (fungi and protists). However, most of the clones showed low identity values, indicating that the majority of sequences might represent novel taxa. Further studies, using a polyphasic approach (*i.e*., including a combination of genotypic and phenotypic approaches) will be necessary to confirm this hypothesis.

**Figure 1:**
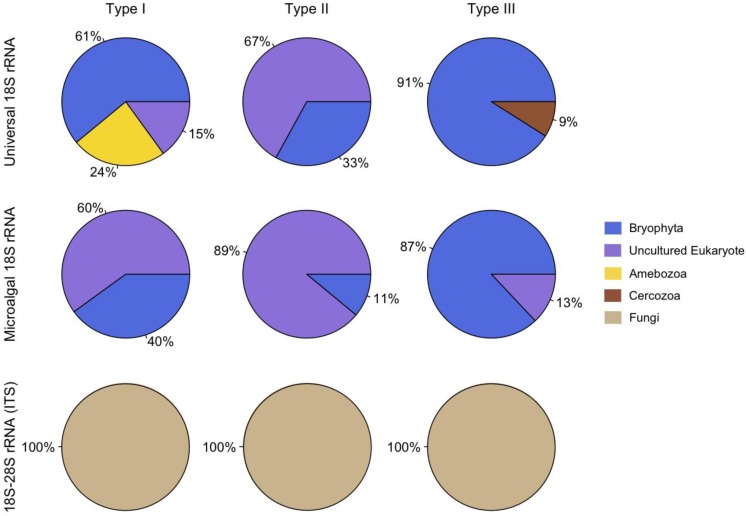
Relative distribution of phylotypes.

The phylotypic abundance data indicates that ascomycetes were present in all three hypolith types, but also possible habitat preferences for certain groups of eukaryotes. For example, amoebozoa were only found in Type I hypolithons (cyanobacteria dominated) whereas cercozoa were present only in Type III hypolithons (moss dominated) (Figure 1). Cyanobacteria can modify the surrounding environment [17], and play critical roles in the structuring of hypolithic communities [12,18]. For example, cyanobacteria produce UV-screening pigments, enzymes, and carotenoids that quench reactive oxygen species, solute-binding materials, water absorbing gels, antifreeze compounds, and ice-nucleating substances [19], which will reduce oxidative, osmotic, freeze-thaw, and dehydration stresses for all organisms embedded within the matrix. In contrast to open soil, hypoliths are also rich in inorganic nutrients, organic carbon and bacteria [18] that may provide substrates for eukaryotic heterotrophs such as protists and the metazoan microfauna. The presence of saprophytic, phagotrophic, parasitic and predatory eukaryotes would increase the inherent capacity for nutrient and energy transfer, thereby increasing trophic complexity and potential resilience to environmental change [12].

**Table 1:** PCR primers used to amplify universal 18S rRNA, Internal Transcribed Spacer (ITS) and microalgal 18S regions of ribosomal RNA genes from eukaryotic microorganisms, and their respective PCR cycling conditions.

Primer Set	Sequence (5’- 3’)	Region of Amplification	PCR Parameters	Reference
EukA	AACCTGGTTGATCCTGCCAGT	18S rRNA gene	94 °C for 3 min; 30 cycles: 94 °C for 45 s, 50 °C for 1 min, 72 °C for 3 min; 72 °C for 20 min	[20]
EukB	TGATCCTTCTGCAGGTTCACCTAC			
ITS1F	CTTGGTCATTTAGAGGAAGTAATC	ITS1-ITS2	94 °C for 5 min; 35 cycles: 94 °C for 1 min, 50 °C for 1 min, 72 °C for 1 min; 72 °C for 20 min	[21,22]
ITS4	CTCCGCTTATTGATATGC			
P45	ACCTGGTTGATCCTGCCAGT	Microalgal 18S	94 °C for 1 min; 37 cycles: 92 °C for 50 s, 57 °C for 50 s, 72°C for 50 s; 72°C for 10 min	[23]
P47	TCTCAGGCTCCCTCTCCGGA	rRNA gene		

Fungal sequences were classified into 13 ascomycete phylotypes (Table 2). Most (86%) were related to the genus Acremonium, while some sequences were affiliated to Stromatonectria and Verrucaria (7% each); although it is worth noting that sequence comparisons of the ITS region gave low similarity values (76%–92%). There have been a limited number of molecular diversity studies of hypolithic [11] and soil [7,24] fungi in the Antarctica, some of which show contrasting results. For example, Fell *et al.* [7] found that both ascomycetes (lichen-forming and decomposers) and basidiomycetes (decomposers and nematode pathogens) were widely distributed in soils, whereas Khan *et al.* [11] reported ascomycetes as the only members in hypolithic fungal communities. This apparent dichotomy in fungal distribution between open soils and hypolithic communities offers a potential line for future research.

Much of the knowledge of fungal communities in Antarctica is based on culture-dependent techniques. To date, over 1,000 non-lichenized fungal species have been recorded [25], including numerous representatives of all of the major fungal groups (ascomycetes, basidiomycetes, zygomycetes and chytrids) but only a single member of the Glomeromycota. The most complete list of Antarctic fungal species known from culturing and collection comprises approximately 68% ascomycetes, 23% basidiomycetes and 5% zygomycetes, with the remaining 4% consisting of oomycetes, chytrids and myxomycetes [26].

Basidiomycetes are commonly associated with old or decaying wood. The complete absence of higher plants in east Antarctica, in particular those with woody components, may represent a significant constraint on the diversity of Antarctic basidiomycetes. Indeed, a recent study has shown that when exotic organic substrates were buried, there was a significant increase in fungal colony-forming units (CFU) in soils in direct contact with the introduced, sterile cellulosic substrates compared to background soil levels [27]. Fungi are often also found in association with bryophyte communities and are thought to exploit the release of dissolved organic C from moss structures due to damage caused by freeze-thaw cycles [28].

The vast majority of Antarctic fungi are mesophiles capable of growing at low temperatures [29]. This, together with their widespread occurrence, could suggest that many Antarctic fungi are particularly resilient cosmopolitan species and therefore likely to be relatively recent colonists [25].

Three OTUs belonging to protists were found (Table 2). Sequences related to amoebozoa showed relatively high homology, 98% and 96%, to members of the genera *Saccamoeba* and *Platyamoeba*, respectively. A cercozoa sequence was closely related to the genus *Cercomonas* (99%). Members of the genera *Sacchamoeba*, *Platyamoeba* and *Cercomonas* have been found previously in Antarctica [30,31] and their abundance and species diversity is greater than that of the nematodes. The protists have generally received much less attention than the bacteria and much of the research on protists is focused on aquatic ecosystems, where these organisms predate bacteria and contribute to the remineralization of major-, minor- and micro-nutrients [32]. In soils, cercozoa and amoeba are known to colonize the pore spaces of soils; however, the smaller pore spaces might provide a more protected or favorable environment with increasing moisture, which might sustain a higher bacterial population and in general, have higher organic matter than a coarser soil fraction with large pore spaces [33]. Amoebae, which are thought to be more resilient than nematodes because of rapid encysting abilities and short life cycle, are thought to be the major predators of bacteria and to make a substantial contribution to carbon and nutrient cycling [34].

Sequences belonging to bryophytes were classified into nine phylotypes (Table 2). In the Antarctic Dry Valleys, bryophytes represent the most “advanced” terrestrial photoautotrophs and are all primary producers providing an additional pathway other than vascular plants for C to enter the soil [35]. However, to our knowledge, there is little information available on hypolithic bryophyte traits. Temperate bryophytes are rich in secondary metabolites such as terpenes and phenolics, which are likely to be allelopathic, affecting decomposition and therefore nutrient cycling [36]. Moreover, mosses can enhance the amount of water infiltrating the soil [37]. Bryophytes do not have stomata and lose water readily from their tissues [38]. They also lack roots, and therefore, they are unable to extract water from depth in a drying soil. Mosses instead depend on the availability of water in the environment, from either humid air, the surface substrate or precipitation. As many species are adapted to survive long periods of desiccation, we suggest that bryophytes may contribute significantly to primary production in periods of moisture sufficiency.

**Table 2:** Affiliation of the clones sampled from hypolithic communities.

Representative Clone	Accession No.	Closest Sequence Match	Accession No.	Identity	Type^a^
Eukaryote 18S rRNA					
Euk75-A1	KC352912	Uncultured *Eucalypta* (Bryophyta)	Y17871	81%	2/I
Euk75-A7	KC352913	Uncultured *Tortula ruralis* (Bryophyta)	AF023682	78%	2/I
Euk75-A8	KC352914	Uncultured *Tortula ruralis* (Bryophyta)	AF023682	86%	2/I
Euk75-A12	KC352915	Uncultured *Tortula ruralis* (Bryophyta)	AF023682	86%	2/I
Euk75-B2	KC352916	Uncultured *Tortula ruralis* (Bryophyta)	AF023682	92%	2/I
Euk75-B9	KC352917	*Saccamoeba limax* (Amoebozoa)	AF293902	98%	2/I
Euk75-C4	KC352918	*Platyamoeba contorta* (Amoebozoa)	DQ229954	96%	2/I
Euk134-C6	KC352919	*Pottia truncata* (Bryophyta)	X95935	99%	4/II
Euk134-D11	KC352920	Uncultured eukaryote	HM490274	100%	4/II
Euk50-B10	KC352921	Uncultured eukaryote	EF024087	91%	5/III
Euk50-D10	KC352922	*Cercomonas plasmodialis* (Cercozoa)	AF411268	99%	5/III
Microalgal 18S RNA					
P50-A4	KC352936	*Mnium hornum* (Bryophyta)	X80985	95%	5/III
P50-B3	KC352937	Uncultured eukaryote	EF024845	99%	5/III
P50-B6	KC352938	*Mnium hornum* (Bryophyta)	X80985	91%	5/III
P50-E7	KC352939	Uncultured eukaryote	EF526889	99%	5/III
P75-E	KC352940	*Bryoxiphium norvegicum* (Bryophyta)	AF223008	85%	2/I
P134-A2	KC352941	Uncultured eukaryote	FN394778	100%	4/II
P134-A11	KC352942	Uncultured eukaryote	HM490274	100%	4/II
IGS 28S-18S rRNA					
ITS65-A1	KC352923	Uncultured *Acremonium* (Ascomycota)	HE977538	82%	1/I
ITS65-A2	KC352924	Uncultured *Acremonium* (Ascomycota)	HE977544	85%	1/I
ITS65-A5	KC352925	Uncultured *Acremonium* (Ascomycota)	HE977544	85%	1/I
ITS65-A8	KC352926	Uncultured *Acremonium* (Ascomycota)	HE977544	78%	1/I
ITS65-B6	KC352927	Uncultured *Acremonium* (Ascomycota)	HE977544	88%	1/I
ITS65-C12	KC352928	Uncultured *Acremonium* (Ascomycota)	HE977544	88%	1/I
ITS65-D2	KC352929	Uncultured *Acremonium* (Ascomycota)	HE977544	87%	1/I
ITS65-D10	KC352930	*Stromatonectria caraganae* (Ascomycota)	HQ112288	80%	1/I
ITS134-A1	KC352931	*Verrucaria* sp. (Ascomycota)	FJ664858	92%	2/II
ITS50-B11	KC352932	Uncultured *Acremonium* (Ascomycota)	HE977544	84%	5/III
ITS50-D2	KC352933	Uncultured *Acremonium* (Ascomycota)	HE977538	80%	5/III
ITS50-E3	KC352934	Uncultured *Acremonium* (Ascomycota)	HE977538	86%	5/III
ITS50-H7	KC352935	Uncultured *Acremonium* (Ascomycota)	HE977538	91%	5/III

^a^Sample no./hypolithon type.

## 3. Experimental Section

### 3.1. Samples Collection

Six hypolith samples from all three Types (2 samples per Type) were obtained from Miers Valley (S78°05.01'–S78°05.921', E163°49.496'–E163°48.149'), Antarctica, in January 2011. The hypolithons were selected and classified as Type I, II or III during sampling. Classification was based on gross morphology of the biomass present on the ventral surfaces of the rocks [8,9]. Samples were recovered aseptically and stored in WhirlPak^®^ bags at 4 °C in the field and during transport. Long term storage was at −80 °C in the laboratory, prior to further analysis.

### 3.2. DNA Extractions and PCR Amplifications

Total soil DNA was extracted using the method described by Von Sigler [39]. Briefly, 1mL of extraction buffer (50 mM NaCl; 50mM Tris-HCl at pH 7.6; 50 mM EDTA; 5% SDS) was added to 1 g of each soil sample in 2 mL vials containing 0.5 g mesh sea-sand. Then, 1 µL of 1M dithiothreitol (DTT) was added and mixed. Samples were shaken for 15 minutes at maximum speed (Vortex Genie 2; Scientific Industries Inc., USA) followed by 3 minutes of centrifugation at 14,000 × g. The supernatant was carefully decanted and 0.5× volumes of chloroform/isoamyl alcohol (24:1) was added to the tubes and mixed. This was followed by centrifugation at 14,000 × *g* for 3 minutes. The aqueous phase was transferred to a 2 mL sterile tube, and an equal volume of chloroform was added, vortexed and centrifuged as before. The aqueous phase was transferred to a sterile 1.5 mL and precipitated using sodium acetate and isopropanol. After centrifugation the pellet was washed by the addition of 70% ethanol, dry and resuspended in 25 µL of sterile distilled water.

The presence of DNA was confirmed by gel electrophoresis on 1% agarose gels, viewed using the AlphaImager 3400 imaging system (Alpha Innotech Co., USA) and quantified using a NanoDrop^®^ ND-1000 UV/Vis Spectrophotometer (NanoDrop Technologies, USA).

The primers and parameters used for the PCR amplifications are described in Table 1. Reactions (25 µL) consisted of ~20 ng metagenomic DNA, 1x DreamTaq™ buffer, 0.2 mM of each dNTP, 0.5 µM of each primer and 0.2 U DreamTaq™ DNA polymerase (Fermentas, USA). PCR products were verified on 1% agarose gels and purified with the GFX™ PCR DNA and Band Purification Kit (GE healthcare, USA) and quantified using the NanoDrop^®^ ND-1000 (NanoDrop Technologies, USA).

### 3.3. Clone Library Construction and Phylogenetic Analysis

Clone libraries were constructed after pooling equal amounts of amplicons from the individual samples for each hypolith type. Aliquots of the pooled products were cloned into *Escherichia coli* GeneHogs™ (Invitrogen) using pGEM-T cloning kit (Promega, USA) and transformants were selected by blue-white screening. The presence of the correctly sized insert was verified by colony PCR using the M13F and M13R vector primers (Fermentas, USA). ARDRA analysis (using *Alu*I and *Hae*III) was used to de-replicate clones. Restriction patterns were visualized on 2% agarose gels and analyzed using Gel-compare II (Applied Maths, Keistraat, Belgium). Plasmid DNA, from a representative of each unique restriction pattern, was extracted with QIAprep Spin Miniprep kit (Qiagen GmbH, Germany) and sequenced using the vector primer M13F with an ABI 3130 DNA Sequencer (Applied Biosystems).

Putative chimeric sequences were filtered using Bellerophon [40]. Sequences of >97% identity (for 18S rRNA amplicons) and >95% (for ITS amplicons) were grouped into OTUs using CD-HIT suite [41]. Taxonomic assignments of representative OTUs were determined by BLAST searches of the NCBI GenBank database (http://www.ncbi.nlm.nih.gov/). Sequences obtained in this study were deposited in the NCBI GenBank database under accession numbers KC352912-KC352942.

## 4. Conclusions

Hypoliths were examined at a single time point using only one molecular approach (*i.e.*, clone libraries). Thus, an in-depth analysis is necessary to elucidate the “true” diversity of the micro-eukaryotes existing in Antarctic hypolithons. However, in spite of its limitations, this baseline study gives insight to the existing micro-eukaryotic community supported by hypoliths in the Miers Dry Valley, Antarctica. We show that these communities are represented by a wide diversity of lower eukaryotes (bryophyta, fungi and protists). The presence of these organisms supports the concept that hypolithic communities constitute complex multi-domain food webs in an environment which is generally considered to be characterised by low diversity and complexity.

The phototrophic bryophyte component is thought to provide a significant contribution to primary productivity in periods of moisture sufficiency. Protists feed on bacterial populations and contribute to standing biomass. Fungi participate in decomposition and recycling, maintaining the balance of nutrients in the discrete and “self-contained” hypolithic microhabitats. The hypothesis that the partitioned activity of co-colonizers may create the necessary conditions for sustained hypolithic productivity is currently being tested in our research group.
